# Epistasis as a Determinant of the HIV-1 Protease's Robustness to Mutation

**DOI:** 10.1371/journal.pone.0116301

**Published:** 2014-12-31

**Authors:** Elena Capel, Mariona Parera, Miguel Angel Martinez

**Affiliations:** Fundació irsiCaixa, Hospital Universitari Germans Trias i Pujol, Badalona, Universitat Autònoma de Barcelona, Barcelona, Spain; Institut Pasteur, France

## Abstract

The robustness of phenotypes to mutation is critical to protein evolution; robustness may be an adaptive trait if it promotes evolution. We hypothesised that native proteins subjected to natural selection *in vivo* should be more robust than proteins generated *in vitro* in the absence of natural selection. We compared the mutational robustness of two human immunodeficiency virus type 1 (HIV-1) proteases with comparable catalytic efficiencies, one isolated from an infected individual and the second generated *in vitro* via random mutagenesis. Single mutations in the protease (82 and 60 in the wild-type and mutant backgrounds, respectively) were randomly generated *in vitro* and the catalytic efficiency of each mutant was determined. No differences were observed between these two protease variants when lethal, neutral, and deleterious mutations were compared (P = 0.8025, chi-squared test). Similarly, average catalytic efficiency (−72.6% and −64.5%, respectively) did not significantly differ between protease mutant libraries (P = 0.3414, Mann Whitney test). Overall, the two parental proteins displayed similar mutational robustness. Importantly, strong and widespread epistatic interactions were observed when the effect of the same mutation was compared in both proteases, suggesting that epistasis can be a key determinant of the robustness displayed by the *in vitro* generated protease.

## Introduction

Genetic robustness is defined as the invariance of phenotypes in the presence of mutations [1], [2]. Proteins can be highly tolerant to single mutations; for example, 84% and 65% of single mutants in bacteriophage T4 lysozyme and the *Escherichia coli lac* repressor, respectively, were previously shown to be functional [3], [4]. We and others previously demonstrated that proteins can also tolerate multiple substitutions [5], [6], [7].

Does mutational robustness favour evolvability? If phenotypes are robust against mutation, a population may have difficulty adapting to environmental change, as several studies have suggested (reviewed in [8]). However, robustness may also increase the amount of neutral genetic variation in a population; if these neutral mutations have epistatic interactions with subsequent mutations (their combined effect on fitness differs from that expected from their effects in isolation), then the number of available phenotypes may be increased in a particular sequence space. Robustness is a form of epistatic interaction because the degree to which genetic variation is expressed depends on the genetic background. Consequently, robustness may allow a population to explore a range of genotypes that may be neutral in one environment but potentially beneficial in another. Recently, neutral diversity in a robust population was shown to accelerate adaptation as long as the number of phenotypes accessible to an individual by mutation was smaller than the total number of phenotypes in the fitness landscape [9]. Human immunodeficiency virus type 1 (HIV-1) proteins, like proteins encoded in other RNA virus genomes, are subjected to a higher mutational burden than cellular proteins due to the error-prone nature of HIV-1 replication. Consequently, in infected individuals, HIV-1 circulates as a quasispecies, that is, as genetically related viruses that are closely distributed around a consensus sequence [10]. This strong mutational pressure suggests that robustness may be an adaptive trait for HIV-1. However, it is still unclear whether RNA viruses have evolved to become robust to mutation.

A seminal evolution experiment demonstrated the evolutionary advantages of neutral mutations by showing that human and bacterial enzymes can acquire new functions without losing their original functions [11]. Mutagenesis-based studies of the cytochrome P450 system also indicated that a protein's capacity to evolve is enhanced by mutational robustness [12]; thermostable variants of cytochrome P450 BM3 accepted a wider range of beneficial mutations. In the same way, several reports suggested that protein robustness is a selectable trait because neutral mutations can be key to future evolutionary innovations [9], [13], [14]. Nevertheless, the precise mechanisms underlying protein robustness are far from being well defined [8], and the adaptive nature of robustness remains to be fully elucidated.

One strategy for exploring protein mutational robustness is to quantify the mutational fitness effects of individual mutations. Mutational fitness effect has been determined for several viral and non-viral proteins by introducing random point mutations into the protein sequence. We previously used this approach to reveal that most mutations have deleterious effects on the HIV-1 protease [15]. Specific processing of viral polypeptides is critical to the replication and maturation of infectious HIV-1 particles as well as a critical target of current antiretroviral treatments [16].

The use of protease inhibitors in HIV-1 therapies is subjecting HIV-1 protease to enormous selective pressure to mutate and evolve, rendering the HIV-1 protease an attractive model system to study evolutionary processes. Here, the mutational robustness of the wild-type HIV-1 protease in reference strain HXB2 (GenBank accession number K03455) was compared to that of an HXB2 mutant, 17a, which harbours four substitutions (I15V, I62V, H69R, and I85V). The 17a protease was generated *in vitro* and chosen for this study because it displayed good catalytic efficiency *in vitro* and *ex vivo* (see below). Strains HXB2 and 17a were subjected to random mutagenesis; 142 individual clones, each carrying one amino-acid substitution, were selected and their catalytic efficiencies were measured. Strikingly, we found that the 17a mutant protease was as robust as the wild-type HXB2 protease to the addition of single, random amino-acid mutations.

## Materials and Methods

### Construction of random HIV-1 protease mutation libraries

Mutagenic polymerase chain reaction (PCR) was carried out in 10 mM Tris-HCl (pH 8.3), 50 mM KCl, 2.5 mM MgCl_2_, 0.5 mM MnCl_2_, 1 µM each oligonucleotide, 5 U Taq polymerase (Invitrogen), and biased deoxynucleoside triphosphate concentrations (300 µM deoxycytidine triphosphate, 1 mM deoxythymidine triphosphate, 300 µM deoxyadenosine triphosphate, 1 mM deoxyguanosine triphosphate; Invitrogen), as previously described [17]. Fifty cycles of 95°C for 30 s and 55°C for 30 s were used, with a final extension at 72°C for 10 min. Input DNA consisted of 1 ng of pBluescript SK plasmid containing wild-type HIV-1 protease DNA (strain HXB2) [18] or mutated HIV-1 protease DNA (strain 17a) per 100 µl of reaction. The PCR oligonucleotides were T3proL (sense, 5′- AATTAACCCTCACTAAAGGGAACAAAAGCTGGAGCTCCACCGCGGTGGCGGCCGCTCTAGAACTAGTGGATCCCCCGGGCTGCAGGAATTCT **TCCTTTAACTTCCCTCAG**-3′, bold indicates the residues of HIV-1 reference clone HXB2, residues 2240–2258; underline indicates an EcoRI restriction site) and T7Xho (antisense, 5′- TAATACGACTCACTATAGGGCGAATTGGGTACCGGGCCCCCCCTCGAG *TCA*A**AGGCCATCCATTCCTGGC** -3′, bold indicates the residues of HIV-1 reference clone HXB2, residues 2588–2604; underline indicates an XhoI restriction site; cursive indicates a stop codon).

The resulting PCR products were digested with EcoRI and XhoI, isolated, and ligated to lambda DNA (Uni-ZAP XR Vector Kit, Stratagene). The ligations were packaged (Uni-ZAP XR Giagapack Cloning Kit, Stratagene), titered, and amplified according to standard procedures. The compositions of the libraries were determined via nucleotide sequencing of the gene encoding the HIV-1 protease in individual phage colonies. Phage DNA from individual colonies was PCR amplified and sequenced with the flanking oligonucleotides T3 (5′-AATTAACCCTCACTAAAGGG-3′) and T7 (5′-TCGAGGTCGACGGTATC-3′) using the ABI PRISM dRhodamine Terminator Cycle Sequencing Kit (Applied Biosystems). Sequence alignment and editing were performed with Sequencer version 4.1 (GeneCodes).

### Determination of protease enzymatic catalytic efficiencies

To determine the enzymatic catalytic efficiency of the identified single-mutation proteases, a phage lambda-based genetic screen was used. This genetic screen is based on the phage lambda regulatory circuit; viral repressor cI is specifically cleaved to initiate the lysogeny-to-lysis switch [19]. Introducing an HIV-1 protease into a wild-type phage cleaves a mutant cI repressor containing a specific HIV-1 protease cleavage site, allowing the phage to undergo lytic replication. As we previously demonstrated, cI repressor cleavage is directly proportional to the catalytic efficiency of the protease [15], [20], [21], [22], [23], [24]. The enzymatic catalytic efficiencies of the HXB2 and 17a single-variant proteases were related to the catalytic efficiencies of the wild-type HXB2 and mutant 17a proteases (100%), respectively. A protease was considered lethal when its catalytic efficiency was significantly indistinguishable from zero, deleterious when its catalytic efficiency was lower than that of the parental protease (100%), neutral when its catalytic efficiency was indistinguishable from parental protease (100%) and beneficial when its catalytic efficiency was higher than that of the parental protease (100%). Briefly, *E. coli* JM109 cells containing plasmid p2X-cI.HIV (HIV-1 protease) were transformed with plasmid pcI.HIV-cro (HIV-1 matrix/capsid cleavage site, amino acids 129–136 of the GAG polyprotein). The resulting cells were grown in the presence of 0.2% maltose, harvested via centrifugation, and resuspended to 2.0 optical density (OD) at 600 nm (OD_600_) in 10 mM MgSO_4_. Cells (200 µl) were infected with 5×10^7^ plaque-forming units of phages expresing the HIV-1 proteases. After 15 min at 37°C, the cells were washed with 1 ml of 10 mM MgSO_4_, harvested via centrifugation, and resuspended in 1 ml of Luria broth containing 12.5 µg tetracycline, 0.2% maltose, 10 mM MgSO_4_, and 0.1 mM isopropyl-beta-D-1-thiogalactopyranoside. The cell cultures were then incubated at 37°C for 3 h and harvested via centrifugation. An additional cycle of selective growth was carried out by resuspending infected cells in a fresh aliquot (200 µl) of JM109 pcI.HIV-cro cells. After two selective growth cycles, the titer of the resulting phage was determined by co-plating the cultures with 200 µl of *E. coli* XL-1 Blue cells (OD_600_ = 2.0 in 10 mM MgSO_4_) on Luria broth plates using 3 ml top agar containing 12.5 µg/ml tetracycline, 0.2% maltose, and 0.1 mM isopropyl-beta-D-1-thiogalactopyranoside. After incubation at 37°C for 6 h, the plaques were counted for growth scores. The catalytic efficiency of each mutant was calculated as the mean ±standard deviation of at least three independent replicates.

### Generation of protease 17a chimeric virus

Recombinant virus containing protease 17a was generated as we described previously [25], [26]. Briefly, HIV-1 protease 17a was amplified from an individual phage clone by PCR using the oligonucleotides 5Prot2HIVPRLSFNF (5′-TCAGAGCAGACCAGAGCCAACAGCCCCACCAGAAGAGAGCTTCAGGTCTGGGGTAGAGACAACAACTCCCCCTCAGAAGCAGGAGCCGATAGACAAGGAACTGTATCCTTTAACTTCCCTCAG-3′, HXB2 residues 2136–2258) and 3Prot2 Xho8R (5′-AATGCTTTTATTTTTTCTTCTGTCAATGGCCATTGTTTAACTTTTGGGCCATCCATTCCTGGC-3′, HXB2 residues 2588–2650). The resulting PCR fragment was 514 bp long and had 90 bp overlap at the 5′ end and 98 bp overlap at the 3′ end with a protease-deleted HXB2 plasmid [27]. One-hundred and fifty nanograms of the PCR product were cotransfected via electroporation with a Bio-Rad GenePulserII instrument into 5×10^6^ MT-4 cells plus 1 µg of a protease-deleted HXB2 clone that had been previously linearised with BstEII. Cell-culture supernatants were harvested when the HIV-1 p24 antigen concentration surpassed 1 µg/ml. Progeny viruses were titrated in CEM-green fluorescent protein (GFP) cells, a Tat-driven GFP-reporter T cell line that expresses GFP when infected by HIV-1 [28]. The 50% cell culture infective dose was calculated according to the method of Reed and Muench [29].

**Figure 1:**
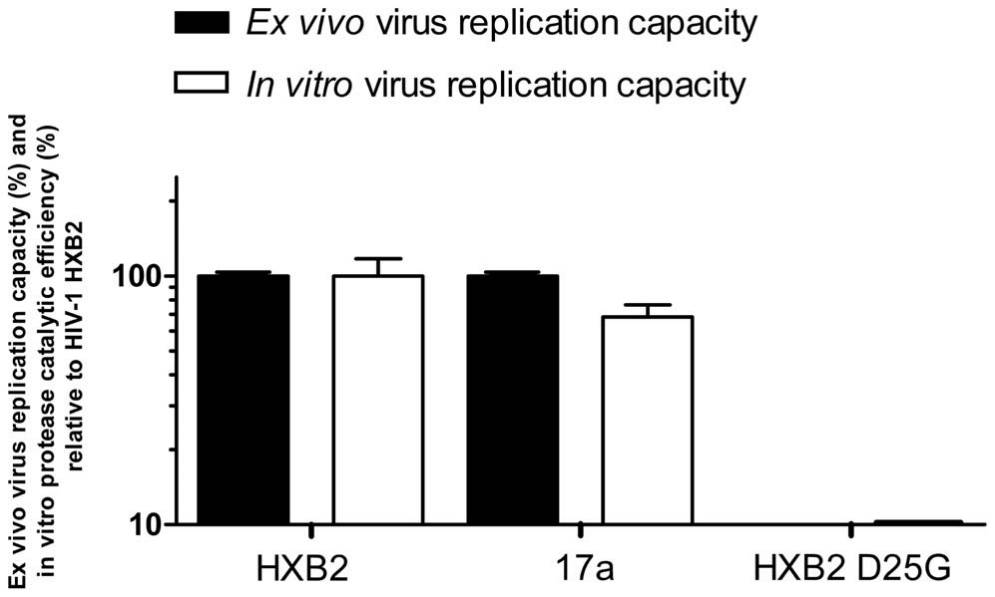
*In vitro* protease catalytic efficiency and *ex vivo* HIV-1 replication capacity of wild-type HXB2 and mutant 17a HIV-1 proteases. Catalytic efficiencies and replication capacities are represented as percentages relative to HIV-1 HXB2 (100%). An HXB2 protease carrying the lethal substitution D25G was also assayed as a negative control. Three independent replicates were performed for each sample. Error bars correspond to standard deviations.

Viral 17a replication capacity was assayed at a multiplicity of infection of 0.01. Aliquots from the culture were harvested 3, 4, 5, 6, and 7 days after infection and fixed in 1% formaldehyde. GFP expression was monitored by flow cytometry (FACSCalibur; BD Biosciences). The slope between days 3 and 7 after infection was calculated for the natural log of the percent of GFP-expressing cells; natural logs are appropriate for exponential growth curves. The replication capacity of strain 17a was compared with that of strain HXB2 (100%; Fig. 1). An HXB2 virus carrying the protease-lethal substitution D25G was also assayed as a negative control.

### Statistical analysis

Statistical analyses consisting of Mann Whitney tests, chi-squared tests and frequency distributions were performed with GraphPad Prism version 4.00 for Windows.

## Results

### Comparable robustness of wild-type (HXB2) and *in vitro* mutated (17a) HIV-1 proteases to single, random amino-acid mutations

**Figure 2:**
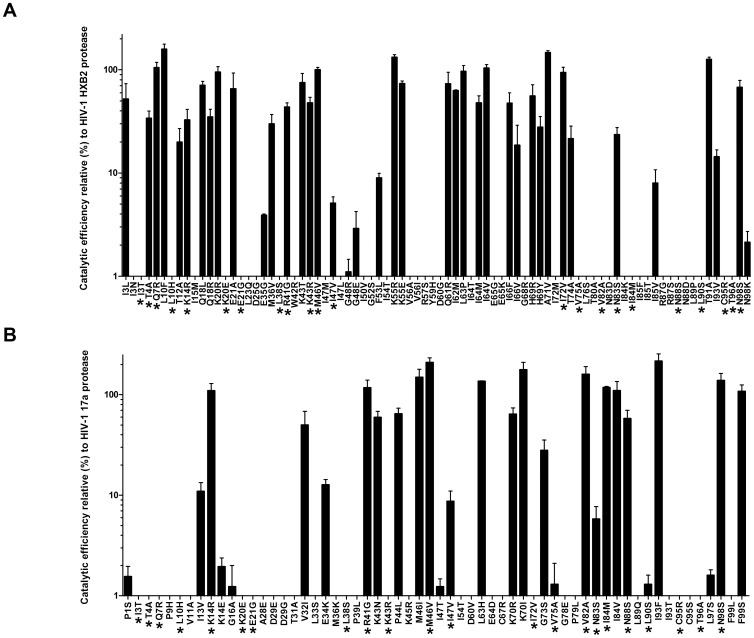
Catalytic efficiencies of HIV-1 protease single mutants. A total of 82 HXB2 single mutants (**A**) and 60 17a single mutants (**B**) of the HIV-1 protease were compared based on HIV-1 matrix (p17)/capsid (p24) protein cleavage. The catalytic efficiency of each protease variant was compared to that of the HXB2 (100%) or the 17a (100%) protease. Substitutions shared by both protease genotypes (HXB2 and 17a) are denoted with asterisks. Three independent replicates were performed for each sample. Error bars correspond to standard deviations.

The wild-type protease used here (HXB2) is derived from the first reported HIV-1 isolate. HXB2 has been used as a prototypic HIV-1 strain in many HIV-1 studies. Protease 17a was derived from HXB2, has four mutations (I15V, I62V, H69R, and I85V), and was selected from a mutant HXB2 protease library generated via random mutagenesis [15]. This mutant protease library, with an average of 2.2 amino-acid mutations per clone, was cloned into phage lambda, and viable mutants (e.g. clone 17a) were selected using a previously described phage lambda-based genetic screen [15]. The 17a protease was chosen for this study because displayed a good *in vitro* and *ex vivo* catalytic efficiencies (Fig. 1) and its amino-acid sequence was not found in the HIV-1 sequence database. In a database of 784 HIV-1 proteases of subtype B (http://www.hiv.lanl.gov), the frequency of the four 17a protease mutations were 0.184, 0.226, 0.001, and 0.001 for I15V, I62V, H69R, and I85V, respectively. When the infrequently occurring H69R and I85V substitutions were individually incorporated into the HXB2 background, H69R was moderately deleterious (65% of wild-type catalytic efficiency) and I85V was highly deleterious (8% of wild-type catalytic efficiency; Fig. 2).

The mutational robustness of the wild-type HXB2 protease was compared to that of the *in vitro*-selected 17a protease, via random mutagenesis and determination of the catalytic efficiencies of individual clones carrying one single amino-acid substitution. Eighty-two clones and 60 clones from the strains HXB2 and 17a, respectively, were isolated and characterised. No significant differences in nucleotide-substitution types were identified between the 82 HXB2 and the 60 17a single mutants (Table 1). The 142 single substitutions were distributed throughout the protein and affected 57 (58%) residues in HXB2 and 50 (51%) residues in 17a (P = 0.5855, chi-squared test; Fig. 2). The HXB2 and 17a mutant libraries shared identical substitutions in 23 residues (Fig. 2).

**Table 1:** Mutation spectra of single mutants in the HXB2 and 17a protease genes.

HXB2, n (%)	17a, n (%)	P value^a^
A→C 5 (6)	A→C 1 (1.5)	0.2123
T→A 5 (6)	T→A 6 (10)	0.4278
T→C 16 (13.5)	T→C 15 (25)	0.5324
A→G 43 (52.5)	A→G 22 (36.5)	0.2510
C→T 3 (4)	C→T 4 (6.5)	0.4372
A→T 4 (5)	A→T 4 (6.5)	0.6665
G→C 1 (1)	G→C 1 (1.5)	0.8257
G→A 5 (6)	G→A 5 (8)	0.6322
C→A 0 (0)	C→A 2 (3)	0.1015

a chi-squared test.

**Figure 3:**
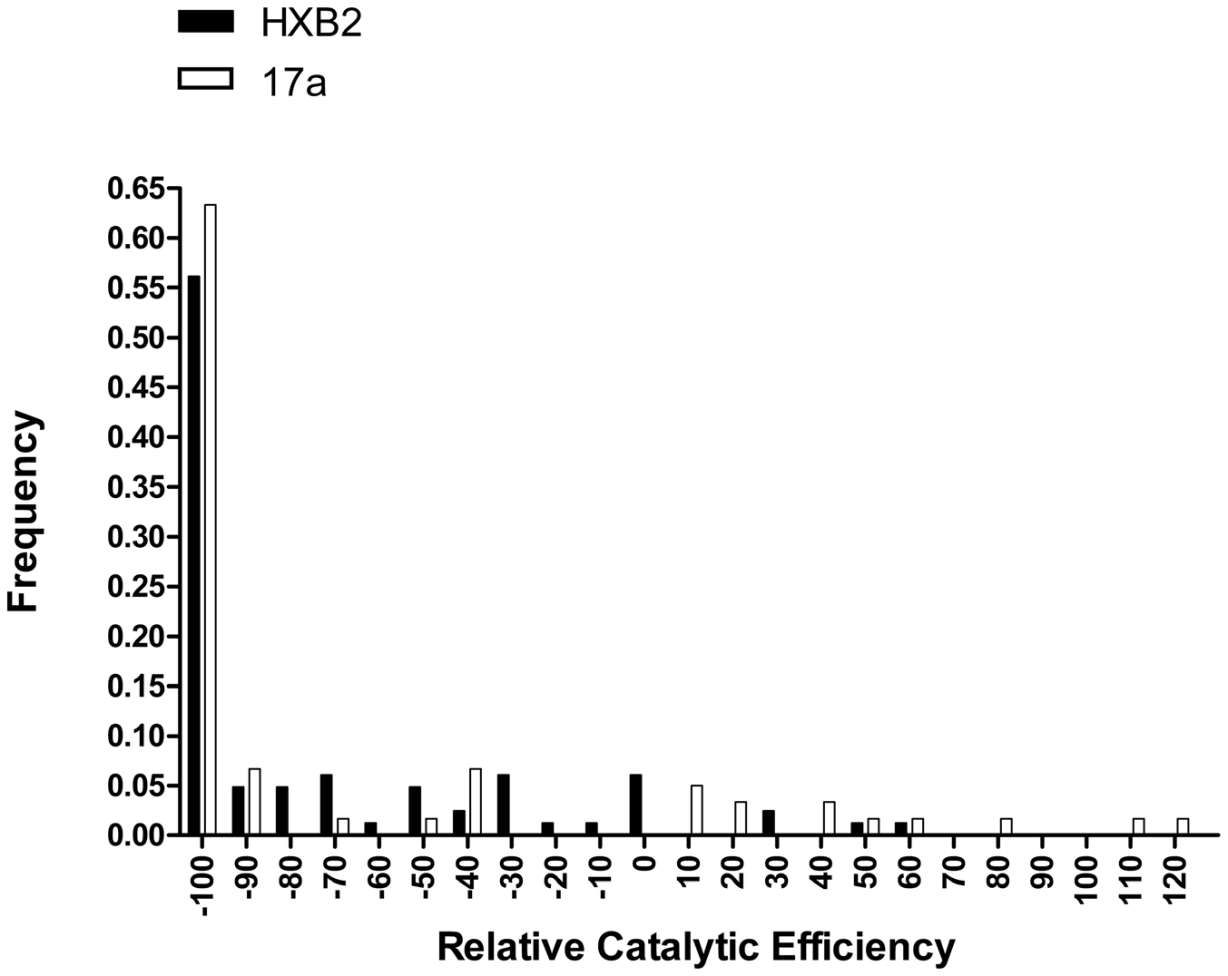
Frequency of catalytic efficiency effects. Relative fitness of 82 HXB2 single mutants (black bars) and 60 17a single mutants (white bars).

The mutational effect was categorized by comparing the catalytic efficiency of the 142 protease mutants with the corresponding starting proteases (HXB2 or 17a) using Mann Whitney tests; each mutated protease was classified as lethal, deleterious, neutral, or beneficial (Fig. 2) (Table 2). The mean catalytic efficiency effects of the 82 HXB2 and 60 17a mutants were −72.6%±4.5 and −64.5%±7.8, respectively. No differences were detected between the mean catalytic efficiency effects of the HXB2 and 17a mutants (P = 0.3414, Mann Whitney test). Similarly, the numbers of lethal, deleterious, neutral, and beneficial substitutions did not differ between HXB2 and 17a (P = 0.8025, chi-squared test). The distributions of the relative catalytic efficiencies of the HXB2 and 17a mutants were both highly skewed toward positive values (g1 = 3.255 and g1 = 4.020, respectively). Both distributions were also highly leptokurtic (i.e. values cluster around the mean) (g2 = 9.573 and g2 = 15.30, respectively) (Fig. 3). The two distributions did not significantly differ from each other (P = 0.2492, Mann Whitney test). Taken together, these data demonstrate that the *in vitro*-selected 17a protease is as robust as the wild-type HXB2 protease to the addition of single, random amino-acid mutations.

**Table 2:** Lethal, deleterious, neutral, and beneficial effects displayed by the 82 HXB2 and 60 17a protease single mutants.

	HXB2		17a	
	Proportion, n (%)	Effect^a^, %	Proportion, n (%)	Effect^a^, %
Lethal	38 (46)	−100	29 (48.3)	−100
Deleterious	29 (35.5)	−74.4	17 (28.3)	−85.6
Neutral	13 (16)	−8.3	12 (20)	21.1
Beneficial	2 (2.5)	94	2 (3.3)	112.8
Total	82 (100)	−72.6	60 (100)	−64.5

a Mean catalytic efficiency effect.

### Intragenic epistasis in the HIV-1 protease

A considerable number of lethal or highly deleterious mutants were distributed among conserved residues that are critical for the structure and function of the protease. In total, 72/142 single-mutant proteases carried a mutation in a conserved region of the enzyme; 10 proteases carried a mutation on the catalytic site (residues 21–32), 21 proteases were mutated on the flap on top of the catalytic site (residues 44–56), 20 proteases harboured a mutation on the substrate-binding site (residues 78–88), and in 21 proteases, the amino- or carboxyl-terminal residues (residues 1–9 and 94–99, respectively), which are involved in protease dimer stabilisation, were mutated. Nevertheless, lethal or deleterious mutations also occurred outside of these preserved coding regions (V11, I15, G16, K20, L33, E35, M36, L38, P39, W42, R57, Y59, D60, I62, I64, E65, R67, G68, I72, T74, V75, L76, L89, L90, and I93).

Six of 15 neutral or beneficial mutations in the HXB2 mutant library (K20R, K43T, L63P, I64V, A71V, and I72V) occurred at positions that were polymorphic in viruses isolated from infected individuals and have been associated with resistance to various HIV-1 protease inhibitors [16], [30]. Analysis of the three-dimensional crystal structure of the HIV-1 protease indicated that most of the neutral or beneficial mutations occurred in peripheral areas of the enzyme, almost entirely in surface loops far from the active site and substrate-binding regions (data not shown). Interestingly, only 2/14 neutral or beneficial 17a mutations, K14R and K70R, were located in known polymorphic positions; these two substitutions are not associated with resistance to protease inhibitors. This observation revealed a property of 17a that is distinct from HXB2 and suggested the presence of intragenic epistatic interactions.

We compared identical amino-acid substitutions in the HXB2 and 17a mutant libraries (Table 3). Mutant interaction was considered epistatic when the same mutations had a significant different effect in the two tested genetic backgrounds (HXB2 or 17a). If intragenic epistasis is absent, we would expect to detect the same catalytic effect across both genetic backgrounds. The same substitution occurred in 23 protease residues, with strong epistatic interactions detected in 12 mutants. The other 11 substitutions rendered HXB2 and 17a lethal or highly deleterious, preventing us from estimating their epistatic interactions. As examples of strong epistasis, Q7R was neutral in the HXB2 background but lethal in the 17a background, I72V was neutral in HXB2 but lethal in 17a, and substitutions V82A and I84M were neutral in 17a but lethal in HXB2 (Table 3). Thus, most of the residues for which epistatic interactions could be determined displayed strong epistasis. Importantly, this epistasis likely causes the 17a protease's robustness, making it as robust as the wild-type HXB2 protease to the addition of single, random amino-acid mutations.

**Table 3:** Catalytic efficiency effects of identical amino-acid substitutions in the HXB2 and 17a mutant libraries.

	Mean catalytic efficiency effect, % ± SE^a^	
Mutation	HXB2	17a
I3T	−100^b^±0.03	−100±0.00
**T4A** c	**−66.1±5.87**	**−100±0.06**
**Q7R**	**4.8±13.28**	**−100±0.03**
L10H	−100±0.15	−100±0.06
**K14R**	**−67.3±8.62**	**9.7±19.80**
K20E	−100±0.00	−100±0.03
E21G	−100±0.03	−100±0.03
L38S	−100±0.00	−100±0.09
**R41G**	**−56.4±4.11**	**17.7±22.98**
**K43R**	**−52.3±6.39**	**−100±0.31**
**M46V**	**0±5.48**	**109.5±23.32**
I47V	−94.9±0.78	−91.3±2.33
I54T	−100±0.02	−100±0.03
**I72V**	**−6±6.19**	**−100±0.03**
V75A	−100±0.07	−98.7±0.80
**V82A**	**−100±0.15**	**59.6±30.65**
**N83S**	**−73.5±4.09**	**−94.2±1.90**
**I84M**	**−100±0.08**	**18.1±2.85**
**N88S**	**−99.2±0.35**	**−42.1±12.15**
L90S	−100±0.02	−98.7±0.31
C95R	−100±0.01	−100±0.18
T96A	−100±0.35	−100±0.21
**N98S**	**−32.3±11.26**	**39±16.92**

a Standard Error (SE).

b A mean catalytic efficiency of −100% represents lethality.

c Residues identified as potentially epistatic are highlighted in bold character.

## Discussion

Different proteins and biological systems display different tolerances to random mutations (reviewed in [31]). However, previous mutational analyses only included wild-type proteins; no study has explored the robustness of *in vitro*-generated protein variant and compared them with wild-type proteins. In this study, we evaluated the robustness of an *in vitro*-generated protein with reference to its wild-type counterpart.

Here, the mutant 17a protease was as vulnerable as the wild-type HXB2 protease to the addition of single, random amino acid mutations. This result is intriguing because if mutational robustness is a heritable trait (i.e., is adaptive), then a wild-type protease should be more robust to mutation than an *in vitro*-generated protease. Our results indicate that the HIV-1 protease is rather fragile in genetic terms; large fractions (46% and 48% for HXB2 and 17a, respectively) of individual, random amino-acid substitutions resulted in lethality. Random single-residue mutagenesis studies reported similar results for native HIV-1 protease (40%) [32] and the HIV-1 protease flap region (amino acids 46–56; 61%) [33]. The HIV-1 capsid coding region was shown to be even more fragile (70%) [31], in contrast to the greater robustness observed in the HIV-1 reverse transcriptase palm subdomain (amino acids 164–203; 28%) [34] and the HIV-1 integrase (35%) [35]. For other viral and non-viral proteins, robustness ranged from 63% (human papillomavirus 16 E1 protein) [36] to 2% (human interleukin-1α protein) [37] (reviewed in [31]).

Notably, to our knowledge this is the first study to investigate the robustness of an *in vitro*-generated protein. Our results provide compelling evidence that *in vitro*-generated proteins may be as robust as wild-type proteins. Nevertheless, it remains to be determined whether HIV-1 protease 17a is as prone to evolution *in vivo* as its wild-type counterpart. Although the 17a protease was as fit as the parental wild-type protease when incorporated into an infectious virus, protease 17a has not been isolated in nature. Critically, we detected many intragenic epistatic interactions in this protein. We previously generated 114 HIV-1 protease genotypes, each carrying pairs of nucleotide-substitution mutations, and determined their separate and combined catalytic efficiency effects in order to systematically identify intragenic epistatic interactions [23]. Although several pairs exhibited significant fitness interactions, including positive and negative epistasis, the average epistatic effect did not significantly differ from zero. However, 40% of pairs created synthetic lethals, which may have biased our results. More recently, we introduced a well-characterised single amino-acid substitution that confers resistance to NS3 protease inhibitors into 56 native hepatitis C virus NS3 protease variants [24]. The introduced amino-acid substitution had different catalytic efficiency effects in different protein variants, providing independent evidence of the role of intragenic epistasis in protein evolution. Similarly, here we detected strong epistasis in 12 HXB2 and 17a mutants carrying the same single amino-acid mutation, including strong antagonistic epistatic interactions.

There is an extensive body of theoretical and empirical considerations of the implications of intragenic epistasis in protein evolution (reviewed in [38], [39], [40], [41], [42], [43], [44]). Both theory and experiment have predicted a tight correlation between robustness and epistasis [45]. Moreover, studies characterising the *in vitro* fitness of clinical isolates of HIV-1 have reported that the fitness landscapes of the HIV-1 protease and reverse transcriptase are characterised by strong epistasis [46], [47]. The epistatic interactions displayed here by the mutant 17a protease strongly suggest that epistasis may underlie the robustness and evolvability of the HIV-1 protease when faced with new, *in vivo* environments (e.g. exposure to protease inhibitors).

We note a limitation of the present study. Although our results are supported by our statistical analysis, we only analysed one mutant background (17a). Since strains HXB2 and 17a have comparable catalytic efficiencies, we cannot discard the previously suggested hypothesis that robustness and fitness are inversely correlated [8]. To clarify this issue, random mutant libraries should be constructed from mutants displaying significant differences in fitness. More experimental work is needed to better define the molecular mechanisms underlying robustness at the protein level.
